# Supplementary material for: Diverse domain architectures of CheA histidine kinase, a central component of bacterial and archaeal chemosensory systems
Source: Microbiol Spectr. 2023 Dec 1;12(1):e03464-23. doi: 10.1128/spectrum.03464-23 (PMC10782961; doi:10.1128/spectrum.03464-23)
Supplement: Table S1 — Domain definitions and IDs. [file spectrum.03464-23-s0001.pdf]

Table S1. Nomenclature and database IDs of CheA domains.

**Common names**	**Pfam domain ID, name**	**Pfam clan ID, name**	**InterPro ID**
P1, histidine phosphotransfer	PF01627, Hpt	None	IPR008207, Signal transduction histidine kinase, phosphotransfer (Hpt) domain
P2, CheY-binding	PF07194, P2<br><br>PF09078, CheY-binding	CL0634, CheY-binding	IPR010808, Chemotaxis protein CheA, P2 response regulator-binding<br>IPR015162, CheY binding
P3, dimerization	PF02895, H-kinase_dim	CL0025, His_Kinase_A	IPR004105, Histidine kinase CheA-like, homodimeric domain
P4, histidine kinase	PF02518, HATPase_c	CL0025, His_Kinase_A	IPR003594, Histidine kinase/HSP90-like ATPase
P5, CheW	PF01584, CheW	None	IPR002545, CheW-like domain
Response regulator, CheY-like	PF00072, Response_reg	CL0304 CheY-like	IPR001789, Signal transduction response regulator, receiver domain
CheC<br>CheX	PF04509, CheC<br>PF13690, CheX	CL0355 CheC-like	IPR007597, CheC-like protein<br>IPR028051, Chemotaxis phosphatase CheX-like domain
